# Lesion Localization and Limb Outcomes in Elderly Patients with and Without Type 2 Diabetes Mellitus Who Undergo Atherectomy-Assisted Endovascular Revascularization due to Symptomatic Peripheral Artery Disease

**DOI:** 10.3390/jcm13216385

**Published:** 2024-10-25

**Authors:** Niki Katsiki, Eva Geiss, Alexander Giesen, Amila Jehn, Christos Rammos, Jan C. Karcher, Christoph Schöfthaler, Grigorios Korosoglou

**Affiliations:** 1 Department of Nutritional Sciences and Dietetics, International Hellenic University, 57400 Thessaloniki, Greece; nikikatsiki@hotmail.com; 2 School of Medicine, European University Cyprus, Nicosia 2404, Cyprus; 3 Cardiology and Vascular Medicine, GRN Hospital Weinheim, 69469 Weinheim, Germany; eva.geiss@grn.de (E.G.); alexander.giesen@grn.de (A.G.); amila.jehn@grn.de (A.J.); christoph.schoefthaler@grn.de (C.S.); 4 Weinheim Cardiovascular Imaging Center, Hector Foundation, 69469 Weinheim, Germany; 5 Department of Cardiology and Vascular Medicine, West German Heart and Vascular Center, University of Duisburg-Essen, 45122 Essen, Germany; christos.rammos@uk-essen.de

**Keywords:** peripheral artery disease (PAD), type 2 diabetes mellitus (T2DM), complex atherosclerotic lesions, major amputation, endovascular treatment, claudication, chronic limb-threatening ischemia (CLTI)

## Abstract

**Background/Objectives:** Type 2 diabetes mellitus (T2DM) represents a major risk factor for peripheral artery disease (PAD). We aimed to evaluate the impact of T2DM on lesion localization and complexity, clinical presentation by Rutherford categories, and limb outcomes in elderly patients with symptomatic PAD undergoing endovascular revascularization. **Methods:** Five hundred consecutive patients with symptomatic infra-inguinal PAD who underwent rotational atherectomy-assisted endovascular revascularization were included. PAD clinical presentation and lesion localization were recorded. The primary endpoints were clinically driven target lesion revascularization (CD-TLR) and major amputation rates during follow-up. **Results:** Overall, 245/500 (49.0%) patients had T2DM, whereas 179 (35.8%) presented with lifestyle limiting claudication and 321 (64.2%) with critical limb-threatening ischemia (CLTI). Median age was 78.0 (IQR = 70.0–84.0) years, and 201 (40.2%) patients were female. The presence of T2DM was significantly more frequent in patients with CLTI vs. those with claudication (58.6 vs. 31.8%; *p* < 0.001). Furthermore, the percentage of patients with below-the-knee (BTK) lesions was significantly higher in patients with vs. without T2DM (40.7 vs. 27.5%, *p* = 0.0002). During median follow-up of 21.9 (IQR = 12.8–28.8) months, CD-TLR rates were similar in patients with vs. without T2DM (HR = 1.2, 95%CI = 0.8–2.0, *p* = 0.39). However, patients with T2DM had a ~5.5-fold increased risk for major above-the-ankle amputation (HR = 5.5, 95%CI = 1.6–19.0, *p* = 0.007). After adjustment for age, gender, lesion complexity, and calcification, T2DM remained predictive for major amputation (*p* = 0.04). **Conclusions:** T2DM is more frequently associated with CLTI, BTK-PAD, and amputations despite successful endovascular revascularization. More stringent surveillance of patients with PAD and T2DM is warranted to prevent atherosclerosis-related complications.

## 1. Introduction

Type 2 diabetes mellitus (T2DM) and lower-extremity peripheral artery disease (PAD) are global health problems with a growing prevalence, being associated with a considerable burden of limb-related and cardiovascular (CV) morbidity and mortality, poor quality of life, as well as high healthcare resource use and costs [1,2,3]. PAD represents a frequent diabetic macrovascular complication, and patients with T2DM have more than 2-fold greater prevalence of PAD compared with the general population [4]. There are several common pathophysiological mechanisms linking T2DM and PAD, including oxidative stress and inflammation. Interestingly, in patients with T2DM, the presence of PAD further increases the risk for cardiovascular (CV) disease and death, as well as for foot morbidity and microvascular complications [5]. When PAD and T2DM co-exist, a significantly greater risk for critical limb-threatening ischemia (CLTI) is noted, which is related with high risk for amputation and death [6].

Based on the above, the prevention or, at least, early diagnosis, followed by adequate treatment of T2DM and/or PAD are of vital clinical importance. Notably, in T2DM patients, a multitargeted intervention is required for preventing PAD development and progression [7,8]. Vice versa, since T2DM is a major risk factor for PAD development [6], PAD patients need to be screened for T2DM. If PAD co-exists with T2DM, further therapeutic strategies are required, involving anti-diabetic treatment, but also potentially selecting certain interventional procedures (according to lesion localization and complexity), as well as a closer follow-up and monitoring of the patients to improve their clinical outcomes and survival.

The aim of the present study was to evaluate the effect of T2DM presence on PAD clinical presentation (by Rutherford categories), lesion localization and complexity, as well as on limb outcomes and survival in elderly patients with symptomatic infra-inguinal PAD undergoing endovascular revascularization. Focus was set on a specific PAD cohort, with complex and calcified lesions, where atherectomy-assisted revascularization was performed.

## 2. Patients and Methods

### 2.1. Study Design and Ethics Disclosures

Our study was conducted in a single vascular center with wide experience with minimal invasive atherectomy-assisted endovascular therapy in complex femoropopliteal and below-the-knee (BTK) lesions [9,10,11,12]. It was designed as a prospective, observational registry. Ethics approval was obtained from the local ethics committee (S-100/2017) of the University of Heidelberg. In addition, the study was registered on the German Clinical Trials Register website (DRKS00016708).

### 2.2. Patient Population

Between July 2017 and February 2022, 1645 consecutive patients with symptomatic PAD due to chronic atherosclerotic lesions and occlusions of peripheral lesions were referred to our center for endovascular treatment. Out of these 1645 patients, 500 required atherectomy-assisted endovascular revascularization, based on our internal standard operating procedures, and were enrolled in our registry [13]. 

CV risk factors, such as hypertension, hyperlipidemia, active smoking, and T2DM, as well as history of CV diseases and current drug treatment, were recorded. Furthermore, ankle-brachial-index (ABI) before and after the endovascular procedures was measured and clinical presentation by Rutherford category was documented. Laboratory data, including serum creatinine, urea, hemoglobin, and high-sensitivity troponin T (hs-TnT) were also evaluated at baseline. Estimated glomerular filtration rate (eGFR) was calculated using the chronic kidney disease epidemiology collaboration (CKD-EPI) creatinine equation [14]. HbA1c values were recorded in patients with T2DM.

All patients included were referred for endovascular treatment either upon clinical assessment or upon recommendation of in-hospital or primary treating vascular specialists. In selected cases, the possible alternative treatment option of open repair was considered and discussed. Typically, patients with CLTI were scheduled for endovascular treatment in due course within 72 h or earlier, depending on clinical symptoms, whereas a time interval of about three months with best medical treatment and exercise preceded the endovascular treatment in patients with lifestyle-limiting claudication.

### 2.3. Endovascular Procedure Protocol

All procedures were performed by 3 experienced interventional cardiologists and angiologists (J.C.K., C.S., and G.K.), specifically trained for these techniques and board-certified by the German Societies of Cardiology and Angiology. In brief, all patients received a bolus of 5.000 I.U. of heparin after placement of a 6F or a 7F sheath introducer in the contralateral or ipsilateral common femoral artery based on lesion localization. Guidewire passage through the lesions or the occlusions was performed from antegrade and, upon failure, the retrograded strategy was used [12,15]. Generally, care was taken to maintain the wire in the intraluminal space throughout the lesion, since atherectomy is generally not recommended by instructions for use (IFU) when entering with subintimal wire passage [10]. During interventional treatment, heparin was additionally injected, aiming for an activated clotting time between 250 and 300 s, which was measured every 30 min after the initiation of the procedure.

The Phoenix atherectomy device and its work principle have already been described previously [10]. It consists of the catheter, which houses the cutter at its distal tip. The cutter rotates at high speed between 10,000 and 12,000 rpm, so that fragmented debris is removed due to strong suction forces, without the need for embolic filter protection. The Phoenix system is delivered over a 0.014-inch guide wire and can be used for the treatment of vessels with a diameter between 2.4 mm and 7 mm. Thus, depending on the vessel diameter, 1.5 mm, 1.8 mm, 2.2 mm, and 2.4 mm Phoenix devices were used. The 1.5 mm (4F) device was used for the treatment of vessels with diameters of 2.0–3.0 mm (typically in BTK, crural, and pedal arteries), the 1.8 mm (5F) device for vessels with diameters of 2.5–4.5 mm (typically in BTK crural arteries), the 2.2 mm (6F) device for vessels with diameters of 3.0–6.0 mm (typically in proximal crural, popliteal, and superficial femoral arteries), and the 2.4 mm (7F) deflecting device for vessels with diameters > 4.5 mm (typically in large popliteal and superficial femoral arteries and in the CFA). No pre-dilation of the lesions was performed prior to the atherectomy procedure. Following atherectomy, lesions were treated with balloon angioplasty or directly by drug coated balloons (DCB). In case of persistent recoil or flow limiting dissections, self-expanding bare metal stents were implanted based on internal standards [12]. Patients were treated medically with a combination of aspirin (100 mg daily) and clopidogrel (75 mg daily) for 1–3 months depending on their ischemic and bleeding risk, as well as lesion localization and complexity [10]. In patients with clinical indication for oral anticoagulation, for example due to atrial fibrillation, oral anticoagulants were combined with clopidogrel (75 mg daily) [16,17].

On the day after the procedure, ABI was measured in all patients, who also received a routine duplex ultrasound to exclude proximal or distal site complications (such as arteriovenous fistula and pseudoaneurysms at the puncture sites) and to assess acute procedural success.

### 2.4. Analysis of Angiographic Data

All angiograms were evaluated for assessment of lesion characteristics by J.C.K. and G.K. as follows: length of the lesion or occlusion, presence or absence of chronic total occlusive (CTO) lesions, lesion classification by the Peripheral Arterial Calcium Scoring System (PACSS) criteria (i.e., grade 0 = no visible calcium; grades 1 and 2 = unilateral calcification < 5 or ≥5 cm, respectively; and grades 3 and 4 = bilateral calcification < 5 or ≥5 cm, respectively) [18], and presence of multi-level PAD based on the Trans-Atlantic Inter-Society Consensus Document on Management of Peripheral Arterial Disease (TASC) classification [19].

### 2.5. Follow-Up and Study Endpoints

Follow-up was performed at regular intervals in our out-patient centers or by the referring physicians. Since surveillance was affected by the COVID-19 pandemic after March 2020 in Germany, missing clinical data were obtained via telephone calls from the patients or the referring physicians. During telephone contact, a standardized questionnaire was used to assess the patient clinical status, re-interventions, major adverse cardiac or limb events, and limiting symptoms. Safety endpoints were perforation, embolization, or access site complications requiring further interventions or open repair during the atherectomy procedure. Procedural endpoints were technical success, defined as residual stenosis < 50% after atherectomy prior to any type of adjunctive treatment [10], procedural success, defined as residual stenosis < 30% after atherectomy and adjunctive treatment, including balloon angioplasty and, if required, stent placement [10], and the need for bail-out stent placement. Primary outcomes were clinically driven target lesion revascularization (CD-TLR) and major above-the-ankle amputations.

### 2.6. Statistical Analysis

Analysis was performed using the commercially available software MedCalc 20.009 (MedCalc software, Mariakerke, Belgium). Continuous variables were expressed as median and interquartile range (IQR) since all were not normally distributed. The Shapiro–Wilk normality test was performed to assess normality vs. skewed distribution. Categorical variables were expressed as proportions and were compared using χ^2^ tests. For comparison of continuous variables, non-parametric tests (Mann–Whitney U-tests or Kruskal–Wallis tests) were performed. Kaplan–Mayer analyses were used to evaluate all-cause mortality, CD-TLR, and major amputation rates over time in patients with claudication vs. CLTI and in patients with vs. without T2DM. Cox-proportional hazard models were performed to evaluate the independent effect of T2DM for the prediction of major amputations. Differences were considered statistically significant at 2-sided *p* < 0.05.

## 3. Results

### 3.1. Demographic Data, Clinical Presentation, and Laboratory Parameters

Among 500 patients, 245 (49.0%) had T2DM, 179 (35.8%) presented with claudication and 321 (64.2%) with CLTI, including 45 (9.0%) patients with resting pain and 276 (55.2%) with wounds. Of 245 patients with T2DM, 57 (23.3%) and 188 (76.7%) presented with claudication vs. CLTI, respectively.

Median age was 78.0 (IQR = 70.0–84.0) years, and 201 (40.2%) patients were female. In patients with T2DM, median HbA1c values were 7.2% (IQR = 6.3–7.9). Patients with T2DM were less frequently female (35.5 vs. 44.7%, *p* = 0.04) and more frequently had CAD (51.4 vs. 37.2%, *p* = 0.001) and history of coronary bypass surgery (14.3 vs. 8.6%, *p* = 0.05) (Table 1). In addition, among patients with claudication (n = 179), 57 (31.8%) had T2DM, whereas 122 (68.2%) did not have T2DM (*p* < 0.0001) (Table 1). In contrast, among patients with CLTI (n = 321), 188 (58.6%) had T2DM and 133 (41.4%) did not (*p* < 0.0001). Thus, the percentage of patients with T2DM was significantly higher among CLTI patients vs. those with claudication (58.6 vs. 31.8%, *p* < 0.0001) (Table 1).

Median serum urea and creatinine were significantly higher in patients with T2DM vs. those without T2DM (42 vs. 36 mg/dL, *p* < 0.001 and 1.12 vs. 0.94 mg/dL, *p* < 0.0001, respectively), whereas median eGFR was significantly lower in patients with T2DM (51.0 vs. 56.0 mL/min/1.73 m^2^, *p* = 0.001). Median levels of hs-TnT were significantly greater in patients with vs. without T2DM (18.8 vs. 13.0 ng/dL, *p* < 0.0001). Furthermore, significantly more patients with T2DM were taking ß-blockers (68.9 vs. 59.2%, *p* = 0.05) and diuretics (51.4 vs. 39.2%, *p* = 0.04) (Table 1).

### 3.2. Lesion Localization, Length, and Calcification

Overall, 614 lesions, including 302 (49.2%) CTOs, were treated by atherectomy-assisted endovascular therapy. Among 614 lesions, 22 (3.6%) were in iliac, 383 (62.4%) in femoropopliteal, and the remaining 209 (34.0%) in BTK arteries (Table 2). Of the 614 treated lesions, 302 referred to patients with T2DM and 312 to patients without T2DM. The percentage of patients with BTK lesions was significantly higher in patients with T2DM (40.7 vs. 27.5%, *p* = 0.0002), whereas significantly more patients without T2DM had femoropopliteal artery lesions (68.3 vs. 56.3%, *p* = 0.0005) (Table 2).

In the total patient population, median lesion and occlusion lengths in femoropopliteal arteries were 17.0 (8.1–26.0) cm and 8.0 (2.8–18.0) cm, respectively; the corresponding values for BTK arteries were 22.0 (14.0–33.0) cm and 12.0 (4.0–22.0) cm, respectively (Table 2). Overall, in the total patient population, lesion calcification was mild (PACSS 0–2) in 121 (24.2%), moderate (PACSS 3) in 175 (35.0%), and severe (PACSS 4) in 204 (40.8%) patients.

Lesion and occlusion lengths, calcification, and lesion complexity (TASC, presence of CTOs) were not statistically different between patients with vs. without T2DM (Table 2).

### 3.3. Technical and Procedural Data

**Figure 1:**
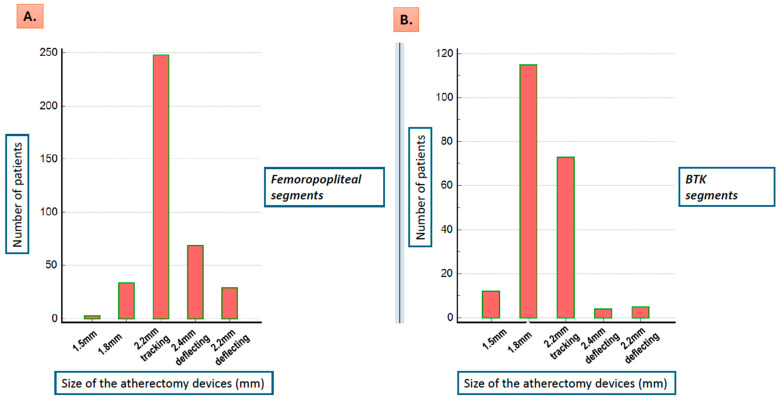
The distribution of the devices used for the treatment of the femoropopliteal (**A**) and the BTK lesions (**B**). BTK indicates below-the-knee.

Among the 500 patients, the 1.5 mm, 1.8 mm, and 2.2 mm tracking and 2.2 mm and 2.4 mm deflecting devices were used in 13 (2.6%), 123 (24.6%), 264 (52.8%), 29 (5.8%), and 71 (14.2%) patients, respectively. The distribution of the devices used in femoropopliteal and BTK lesions is shown in Figure 1A,B.

Technical and procedural success rates were 60.0% (300 patients) and 99.6% (498 patients), respectively. Atherectomy-associated perforations occurred in 5 (1.0%) patients; 3 of the cases were managed by prolonged balloon inflation, whereas 2 cases required the implantation of a covered stent. Peripheral embolization occurred in 11 (2.2%) patients, which were all managed by using catheter aspiration, without persistent vessel occlusions, clinical symptoms, or the need for local lysis. Procedural success rates and complications were not significantly different in patients with vs. without T2DM (*p* = 0.17 and 0.94, respectively).

For femoropopliteal lesions (n = 383), DCB was used in 372 (97.1%) cases. The mean number of DCBs for femoropopliteal lesions was 2.0 (2.0–3.0). Stent or covered stent implantation was necessary in 68 (17.7%) femoropopliteal lesions. Regarding BTK lesions (n = 209), DCB was used in 50 (23.9%) cases, while the remaining were treated using uncoated balloon angioplasty. Bail-out stenting was performed in 13 (6.2%) cases.

**Figure 2:**
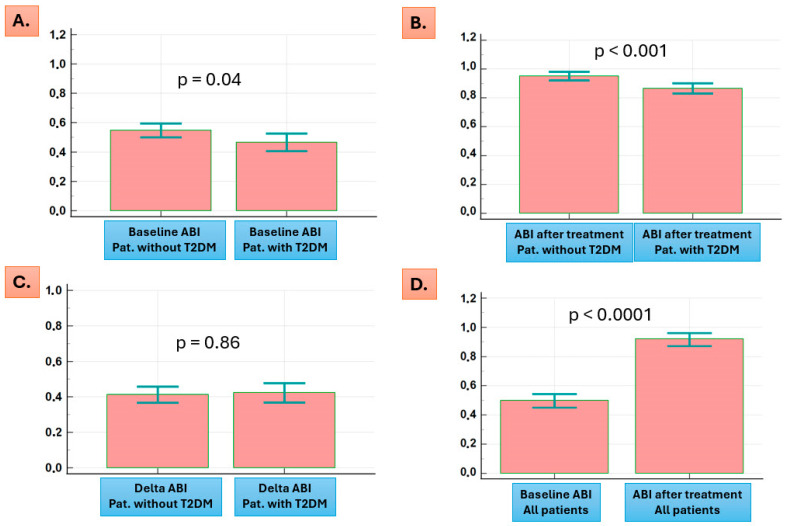
Patients with T2DM had lower ABI at baseline (**A**) and after endovascular revascularization (**B**). Increases in ABI by endovascular treatment were not statistically significant between patients with and without T2DM (**C**). Significant increases in ABI were observed after revascularization in all patients (**D**). T2DM indicates type 2 diabetes mellitus, and ABI indicates ankle-branchial-index.

Patients with T2DM had lower ABI both at baseline (0.45 (IQR = 0.36–0.61) vs. 0.54 (IQR = 0.41–0.65), *p* = 0.04) and after the endovascular treatment (0.86 (IQR = 0.78–0.96) vs. 0.97 (IQR = 0.87–1.02), *p* < 0.001) (Figure 2A,B). Median differences in ABI were, however, not statistically significant between patients with and without T2DM (0.40 (IQR = 0.28–0.51) vs. 0.40 (IQR = 0.31–0.52), *p* = 0.86) (Figure 2C). Significant increases in ABI were observed after revascularization in all patients (0.50 (IQR = 0.40–0.65 vs. 0.92 (IQR = 0.82–1.00), *p* < 0.001)) (Figure 2D).

### 3.4. Clinical Endpoints

**Figure 3:**
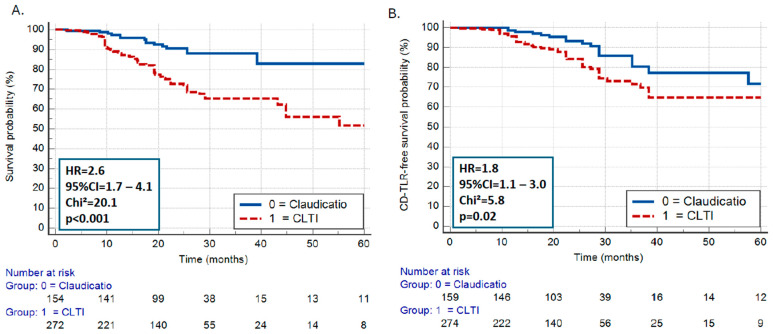
All-cause mortality (**A**) and CD-TLR rates (**B**) in patients with CLTI vs. those with claudication. CD-TLR indicates clinically driven target lesion revascularization; CLTI indicates critical limb threatening ischemia.

Follow-up information of ≥6 months was available in 436 (87.2%) patients during a median time of 21.9 (IQR = 12.8–28.8) months. During this time, 115 (23.0%) patients died, 65 (13.0%) underwent CD-TLR, and 10 (2.0%) patients had major amputation. All-cause mortality and CD-TLR rates were both significantly higher in patients with CLTI vs. those with claudication [hazard ratio (HR) = 2.6, 95%CI = 1.7–4.1, *p* < 0.001 and 1.8, 95%CI = 1.1–3.0, *p* = 0.02, respectively] (Figure 3A,B).

**Figure 4:**
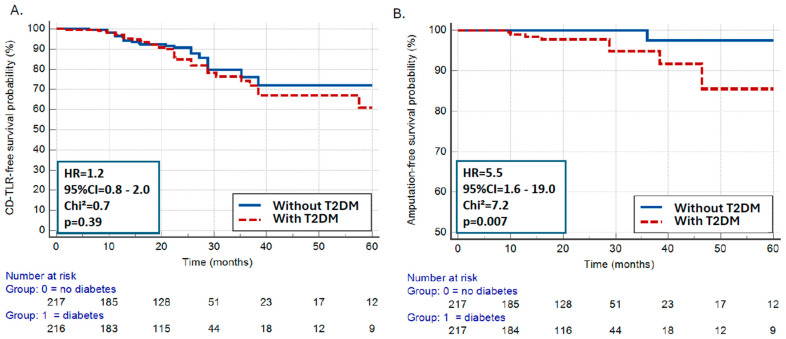
CD-TLR (**A**) and all-cause mortality rates (**B**) in patients with vs. without T2DM. CD-TLR indicates clinically driven target lesion revascularization.

CD-TLR rates were similar in patients with vs. without T2DM (HR = 1.2, 95%CI = 0.8–2.0, *p* = 0.39) (Figure 4A). Similar results were present after differentiation in patients with claudication vs. CLTI (Supplementary Figure S1).

However, patients with T2DM had a ~5.5-fold increased risk for major above-the-ankle amputation during follow-up in comparison to patients without T2DM (HR = 5.5, 95%CI = 1.6–19.0, *p* = 0.007) (Figure 4B). No difference in all-cause mortality was noted in patients with vs. without T2DM (HR = 1.3, 95%CI = 0.9–2.0, *p* = 0.16).

After adjustment for age, gender, lesion complexity, and lesion calcification, Cox-proportional hazard model analysis showed that T2DM remained an independent predictor of major amputation during follow-up (*p* = 0.04) (Table 3).

## 4. Discussion

In the present prospective, observational study, we evaluated the effect of T2DM presence on PAD clinical presentation (by Rutherford categories), lesion localization and complexity, as well as on limb outcomes and survival among 500 elderly patients with symptomatic infra-inguinal PAD undergoing endovascular revascularization. Overall, 245 (49.0%) had T2DM, whereas 179 (35.8%) presented with claudication and 321 (64.2%) with CLTI. Of 245 patients with T2DM, 57 (23.3%) and 188 (76.7%) presented with claudication vs. CLTI, respectively. Patients with T2DM were less frequently female compared with patients without T2DM and more frequently had a history of CAD and coronary bypass surgery. The increased prevalence of CAD and coronary bypass surgery observed in patients with T2DM (vs. those without T2DM) is in line with the previous literature showing that patients with T2DM are more likely to have a history of cardiac diseases.

Regarding laboratory data, patients with T2DM had more advanced chronic kidney disease (CKD), assessed by eGFR, compared with patients without T2DM. Indeed, CKD is a common diabetic microvascular complication, and thus patients with T2DM are more prone to CKD development and progression [20,21]. Of note, due to the frequent co-existence of T2DM with both CAD and CKD, as well as with non-alcoholic fatty liver disease [22], now referred to as metabolic dysfunction-associated steatotic liver disease [23,24], T2DM is currently regarded as a Cardiac-Kidney-Liver (CKL) syndrome [25], thus highlighting the need for a holistic cardio-reno-metabolic therapeutic approach in these patients [8,26,27]. In the present study, patients with T2DM had higher median levels of hs-TnT in comparison to patients without T2DM. This finding is in accordance with previous studies reporting greater hs-TnT concentrations in patients with vs. without T2DM in different clinical settings, such as heart failure [28] and acute myocardial infarction [29]. Of note, in patients with PAD (with or without T2DM), hs-TnT levels may independently predict limb and CV outcomes [30,31,32].

Regarding PAD features and outcomes, in the present study, the percentage of patients with T2DM was significantly higher among CLTI patients vs. those with claudication. Indeed, T2DM has been linked with a more severe form of PAD. For example, a prospective population-based study (n= 92,728 participants, 59.3% being ≥75 years) reported that T2DM was associated with a greater risk for incident PAD events (risk ratio, 3.01; 95% CI, 1.69–5.35; *p* < 0.001) [33]. T2DM was also significantly more prevalent in patients with CLTI compared with acute limb ischemia and acute visceral ischemia events (44.1% vs. 12.9% vs. 11.5%; *p* < 0.001, respectively) [33]. Similarly, others have showed that patients with CLTI and T2DM are at a higher risk for amputation compared to those without T2DM [34]. Of note, T2DM may increase the incidence and severity of limb ischemia by approximately 2- to 4-fold [35].

Although lesion and occlusion lengths, CTO lesions, lesion calcification, and lesion complexity, as well as CD-TLR rates were similar between patients with vs. without T2DM in the present study, patients with T2DM had a ~5.5-fold increased risk for major above-the-ankle amputation during follow-up. After adjustment for age, gender, lesion complexity, and lesion calcification, Cox-proportional hazard model analysis showed that T2DM remained an independent predictor of major amputation during follow-up. Previous studies have also reported an increased risk for amputation in patients with vs. without T2DM. For example, in a previous prospective, population-based cohort study involving 63,134 middle-aged or older participants in Singapore, T2DM patients had a 13-fold higher risk of lower-extremity amputation for CLTI compared with patients without T2DM (HR, 13.61; 95% CI 11.64–15.91), irrespective of other traditional vascular risk factors (e.g., obesity, age, gender, smoking, alcohol consumption, hypertension, history of CAD, or stroke) [36]. Another review reported that, among PAD patients, 25% to 90% of amputations are related to T2DM [37]. Several factors may contribute to this risk, including the combination of impaired arterial flow (due to PAD), peripheral neuropathy and infection (due to T2DM), as well as the presence of diabetic ulcers, foot deformities, and impaired wound healing [37]. Other reports also highlight the fact that T2DM is related to a lower limb survival among patients with CLTI [34], while in elderly patients (i.e., aged 65 to 74 years), T2DM may further raise the risk of amputation up to more than 20-fold [35]. In a recent study, however, younger patients with diabetes undergoing non-elective revascularization due to CLTI exhibited a higher risk for major amputation [38].

In the present study, atherectomy-assisted endovascular treatment exhibited good safety with low CD-TLR and amputation rates (13.0% and 2.0%, respectively) in complex and calcified femoropopliteal and BTK lesions. Especially in patients with diabetes and increased intimal and medial calcification, the use of dedicated vessel preparation tools such as atherectomy may be effective for lesion preparation, by improving vessel compliance without causing barotrauma [13]. In addition, procedural success rates were high (>99%). Atherectomy-related complication rates, including perforation and peripheral embolization, were low and could all be managed by endovascularly, without the need for conversion to open surgery. Recently, the rate of peripheral embolization was shown to be higher with atherectomy vs. non-atherectomy-assisted endovascular procedures in a retrospective multi-center registry [16]. In this study, different atherectomy types and devices, including directional and different rotational devices, were involved. In our study, using the Phoenix device and without distal embolic protection, the rate of peripheral embolization was low (2.2%). However, attention is needed with peripheral embolization, which may adversely affect future limb outcomes, especially in patients with T2DM and compromised microvascular function. Thus, more in depth analysis is required in future studies.

Regarding the adjunct treatment of femoropopliteal lesions, 97.1% of the lesions were treated with DCB, and stent placement was necessary in 17.7% of the cases, which is relatively low considering the lesion calcification and complexity. BTK lesions, on the other hand, were mostly treated with atherectomy combined with balloon angioplasty, whereas stent placement was necessary only in 6.2% of the cases. Notably, the 2.2 mm and 2.4 mm atherectomy devices were used for the treatment of femoropopliteal arteries, whereas for BTK vessels, the 1.8 mm device was most frequently used. Importantly, procedural success rates, CD-TLR rates, and complications were not significantly different in patients with vs. without T2DM. This agrees with previous observations showing that PAD can be treated successfully with endovascular interventions or bypass surgery in patients both with and without T2DM [17,39].

### Limitations

The present study has some limitations. First, this study was conducted in a single vascular center and the number of patients was relatively low. In addition, the findings apply only to patients with especially complex and calcified lesions who underwent atherectomy-assisted endovascular treatment, so the results cannot be generalized in patients with less complex and calcified lesions. Second, blood pressure and lipid values were not recorded prior to vascular procedures, as well as T2DM drug therapy. These data may have affected the findings, and this should be considered when interpreting the results. Of note, median HbA1c values were 7.2% in T2DM patients; the HbA1c goal is usually <7%, but the glycemic target for each individual patient may be more or less stringent, according to several factors such as life expectancy, established vascular complications, comorbidities, risks related to hypoglycemia and other drug adverse effects, disease duration, individual needs, and preferences [40]. We also need to consider that the present study included elderly patients. However, we did not examine the effect of glycemic control in lesion localization and complexity, clinical presentation by Rutherford categories, and limb outcomes. Finally, data on vessel patency based on duplex ultrasound and serial ABI measures are not available. However, CD-TLR is also an important clinical endpoint, since repeated interventions are associated with higher health costs, worse outcomes, and low quality of life. Strengths of the study are its prospective design and a clinically significant follow-up time (median: 21.9 months). In addition, to the best of our knowledge, this is the largest study until now, reporting on the safety, effectiveness, and outcomes of elderly patients with and without T2DM, undergoing atherectomy-assisted endovascular revascularization in an all-comer population with PAD due to atherosclerotic disease, localized in multiple femoral, popliteal, and BTK segments.

## 5. Conclusions

Rotational atherectomy exhibits an excellent safety profile for the treatment of patients with complex and calcified femoropopliteal and BTK lesions with low rates of stenting and clinically acceptable CD-TLR rates. Patients with T2DM were more likely to present with BTK than femoropopliteal artery involvement and with CLTI rather than claudication. Despite similar procedural success, complication, and CD-TLR rates in patients without vs. with T2DM, the latter exhibit significantly higher major amputation rates during follow-up. Therefore, it is of clinical importance to prevent T2DM among PAD patients, or to at least diagnose it early and treat it adequately, as well as to monitor PAD patients with T2DM closely in relation to limb and other outcomes.

## Figures and Tables

**Table 1:** Demographic data, clinical presentation, laboratory parameters, and procedural data of all patients, as well as patients with and without T2DM.

Demographic Data, Clinical Presentation, Laboratory Parameters, and Procedural Data	All Patients (n = 500)	Patients with T2DM (n = 245)	Patients without T2DM (n = 255)	*p*-Values
	Demographic data			
Age (years)	78.0 (70.0–84.0)	78.0 (70.0–83.0)	79.0 (69.0–84.0)	0.30
Female gender, n (%)	201 (40.2%)	87 (35.5%)	114 (44.7%)	0.04
Arterial hypertension, n (%)	482 (96.4%)	238 (97.1%)	244 (95.7%)	0.37
Hyperlipidemia, n (%)	440 (88.0%)	221 (90.2%)	219 (85.9%)	0.14
Active or former smoking, n (%)	210 (42.0%)	95 (38.8%)	115 (45.1%)	0.15
History of CAD, n (%)	221 (44.2%)	126 (51.4%)	95 (37.2%)	0.001
History of myocardial infarction	97 (19.4%)	56 (22.9%)	41 (16.1%)	0.06
History of coronary bypass surgery	57 (11.4%)	35 (14.3%)	22 (8.6%)	0.05
History of atrial fibrillation	140 (28.0%)	76 (31.0%)	64 (25.1%)	0.17
	PAD clinical presentation by Rutherford categories			
Rutherford category 2–3 (claudication)	179 (35.8%)	57 (23.3%)	122 (47.8%)	<0.0001 *
Rutherford category 4 (resting pain)	45 (9.0%)	18 (7.3%)	27 (10.6%)	
Rutherford category 5 (wound healing/tissue loss)	276 (55.2%)	170 (69.4%)	106 (41.6%)	
	Drug therapy			
Dual platelet therapy after treatment	306 (61.2%)	144 (58.8%)	162 (63.5%)	0.58
Oral anticoagulants	158 (31.6%)	77 (31.4%)	81 (31.8%)	0.94
ACE inhibitors or AT2 receptor blockers	214 (42.8%)	107 (43.7%)	107 (41.9%)	0.80
ß-Blockers	320 (64.0%)	169 (68.9%)	151 (59.2%)	0.05
Diuretics	226 (45.2%)	126 (51.4%)	100 (39.2%)	0.04
Statins	463 (92.6%)	226 (92.2%)	237 (92.9%)	0.59
	Laboratory parameters			
Creatinine (mg/dL)	1.01 (0.83–1.39)	1.12 (0.87–1.57)	0.94 (0.80–1.18)	<0.0001
eGFR (mL/min/1.73 m^2^)	54.0 (44.3–74.8)	51.0 (35.0–68.8)	56.0 (44.3–74.8)	0.001
Urea (mg/dL)	39.0 (29.0–53.0)	42 (33.0–64.3)	36 (27.0–49.2)	<0.001
Hemoglobin (mg/dL)	12.8 (11.1–14.1)	12.7 (10.8–14.0)	13.0 (11.4–14.3)	0.05
High-sensitivity troponin T (ng/L)	14.1 (9.3–23.3)	18.8 (11.9–30.0)	13.0 (8.3–19.7)	<0.0001
HbA1c (%)	NA	7.2 (6.3–7.9)	NA	NA
	Procedural data			
Contrast agent administration (mL)	120.0 (90.0–160.0)	110.0 (80.0–150.0)	120.0 (100.0–162.5)	0.02
Procedural duration (min)	67.5 (51.0–89.0)	62.0 (48.3–84.0)	70.0 (54.0–93.8)	0.006
Radiation exposure (cGY × cm^2^)	1254 (542–3469)	1056 (496–3748)	1328 (581–3342)	0.39

PAD, peripheral artery disease; CAD, coronary artery disease; ACE, angiotensin-converting enzyme; AT2, angiotensin 2; eGFR, estimated glomerular filtration rate; HbA1c, glycated hemoglobin; NA, not applicable. * *p*-value comparing all Rutherford categories in patients with vs. without T2DM.

**Table 2:** Lesion localization and characteristics in all patients and patients with and without T2DM.

	All Patients (n = 500)	Patients with T2DM (n = 245)	Patients without T2DM (n = 255)	*p*-Values
	Lesion localization			
Target lesions	n = 614	n = 302	n = 312	
Iliac artery lesions	22 (3.6%)	9 (3.0%)	13 (4.2%)	0.44
Femoropopliteal artery lesions	383 (62.4%)	170 (56.3%)	213 (68.3%)	0.0005
Below-the-knee (BTK) artery lesions	209 (34.0%)	123 (40.7%)	86 (27.5%)	0.0002
	Lesion length and presence of occlusion			
Lesion length (cm) in femoropopliteal lesions	17.0 (8.1–26.0)	18.0 (10.0–26.0)	16.0 (8.0–26.0)	0.24
Lesion length (cm) in BTK lesions	22.0 (14.0–33.0)	22.0 (13.2–32.0)	22.0 (14.0–35.0)	0.68
Occlusion length (cm) in femoropopliteal lesions	8.0 (2.8–18.0)	6.0 (2.0–18.0)	8.9 (4.0–18.0)	0.10
Occlusion length (cm) in BTK lesions	12.0 (4.0–22.0)	8.0 (4.0–22.0)	12.0 (5.8–21.5)	0.49
Patients with at least 1 total occlusion	278 (55.6%)	127 (51.8%)	151 (59.2%)	0.10
Number of CTO lesions per patient	1.0 (0–1–0)	1.0 (0–1–0)	1.0 (0–1–0)	0.13
	Lesion calcification by PACSS			
PACSS score 0–2, number of patients (%)	121 (24.2%)	54 (22.0%)	67 (26.3%)	0.87
PACSS score 3, number of patients (%)	175 (35.0%)	98 (40.0%)	77 (30.2%)	
PACSS score 4, number of patients (%)	204 (40.8%)	93 (38.0%)	111 (43.5%)	
	Lesion complexity by TASC			
TASC A/B, number of patients (%)	94 (18.8%)	54 (22.0%)	40 (15.7%)	0.19
TASC C, number of patients (%)	289 (57.8%)	136 (55.5%)	153 (60.0%)	
TASC D, number of patients (%)	117 (23.4%)	55 (22.5%)	62 (24.3%)	

CTO, chronic total occlusive; PACSS, Peripheral Arterial Calcium Scoring System; TASC, Trans-Atlantic Inter-Society Consensus Document on Management of Peripheral Arterial Disease.

**Table 3:** Cox-proportional hazard model analysis for the prediction of major amputations.

	Coefficient	Standard Error	Wald	Hazard Ratios	95% Cl	*p*-Values
Age	0.012	0.033	0.14	1.01	0.94 to 1.08	0.70
Gender	−0.64	0.73	0.77	0.52	0.12 to 2.20	0.37
T2DM	2.22	1.07	4.31	9.29	1.13 to 76.19	0.04
TASC classification	−0.041	0.52	0.006	0.95	0.34 to 2.69	0.93
Calcification by PACSS	−0.30	0.38	0.64	0.73	0.34 to 1.55	0.42

SE, standard error; PACSS, Peripheral Arterial Calcium Scoring System; TASC, Trans-Atlantic Inter-Society Consensus Document on Management of Peripheral Arterial Disease.

## Data Availability

The raw data supporting the conclusions of this article will be made available by the authors on request.

## Supplementary Materials

The following supporting information can be downloaded at: https://www.mdpi.com/article/10.3390/jcm13216385/s1, Figure S1: CD-TLR rates in patients with lifestyle limiting claudication (A) versus CLTI (B), based on the presence versus absence of T2DM. CD-TLR, indicates clinically driven target lesion revascularization.
