# Supplementary material for: Lesion Localization and Limb Outcomes in Elderly Patients with and Without Type 2 Diabetes Mellitus Who Undergo Atherectomy-Assisted Endovascular Revascularization due to Symptomatic Peripheral Artery Disease
Source: J Clin Med. 2024 Oct 25;13(21):6385. doi: 10.3390/jcm13216385 (PMC11546110; doi:10.3390/jcm13216385)
Supplement: Supplementary file 1 [file jcm-13-06385-s001.zip › jcm-3229865-supplementary.pdf]

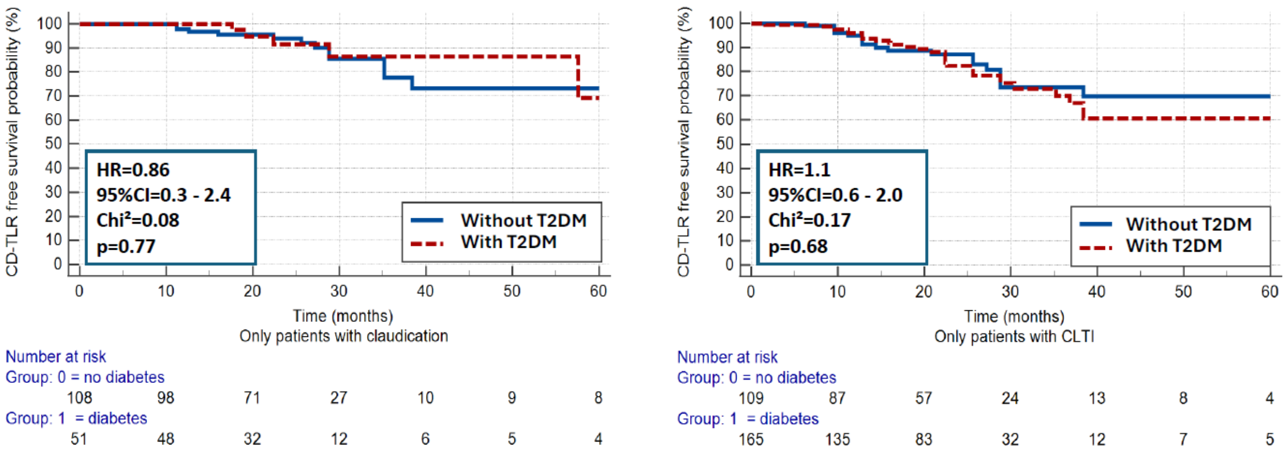

A B

Figure S1. CD-TLR rates in patients with lifestyle limiting claudication (A) versus CLTI (B), based on the presence versus absence of T2DM. CD-TLR, indicates clinically driven target lesion revascularization.
